# Effect of Corrosive Environment on the High-Cycle Fatigue Behavior of Reinforced Concrete by Epoxy Resin: Experimental Study

**DOI:** 10.3390/polym15193939

**Published:** 2023-09-29

**Authors:** Kazem Reza Kashyzadeh

**Affiliations:** Department of Transport, Academy of Engineering, RUDN University, 6 Miklukho-Maklaya Street, Moscow 117198, Russia; reza-kashi-zade-ka@rudn.ru

**Keywords:** conventional concrete, polymer concrete, corrosive environment, axial fatigue test, S-N curve

## Abstract

Large engineering structures made of various materials, including concrete (e.g., bridges, dams, buildings, and multilevel car parks), steel (e.g., power towers, ships, and wind turbines), or others, are often subjected to severe vibration, dynamic, and cyclic loads, which lead to crack initiation, crack growth, and finally structural failure. One of the effective techniques to increase the fatigue life of such structures is the use of reinforced materials. In the meantime, environmental factors, such as corrosion caused by corrosive environments, also affect the fatigue behavior of materials. Therefore, the main purpose of this paper is to study the influence of corrosive environment on the high-cycle fatigue (HCF) behavior of concrete reinforced by epoxy resin. For this purpose, five corrosive environments with different intensities, including fresh air, water: W, sea water: SW, acidic: AC, and alkaline: AL, were considered and the laboratory samples of conventional concrete (CC) and polymer concrete (PC) were immersed in them for one month. Next, axial fatigue tests were performed under compressive–compressive loading with a frequency of 3 Hz on cylindrical specimens. Moreover, to achieve reliable results, for each stress amplitude, the fatigue test was repeated three times, and the average number of cycles to failure was reported as the fatigue lifetime. Finally, the stress–life cycle (S-N) curves of different states were compared. The results showed that polymer concrete can resist well in corrosive environments and under cyclic loads compared to the conventional concrete, and in other words, the epoxy resin has performed its task well as a reinforcer. The results of fatigue tests show that the load bearing range of 10 tons by CC has reached about 18 tons for PC, which indicates an 80% increase in fatigue strength. Meanwhile, the static strength of samples in the vicinity of fresh air has only improved by 12%.

## 1. Introduction

Most old building structures are made of conventional concrete, and are currently either at the end of their life, have been replaced with new structures according to the technological advancements, or need minor repairs [1,2]. Therefore, it is necessary to evaluate the remaining life of these materials, e.g., concrete, in real working conditions with the aim of deciding on their status and related future plans [2]. Meanwhile, there are concrete structures such as marine platforms and integrated concrete bridges, etc., which are installed in water [3,4]. Although some scholars believe that fatigue is the most destructive phenomenon and the main cause of component failure in various industries [5,6], in some cases, environmental factors and harsh working conditions, such as corrosive environment, can lead to more damage in combination with the fatigue phenomenon. In other words, corrosion–fatigue is more dangerous than each of the phenomena separately [7,8]. However, results of previous research show that each of the phenomena of fatigue and corrosion alone is destructive and can lead to the complete failure of a large structure. Hence, the main focus of this article is on evaluating the fatigue behavior of concrete in various corrosive environments with different pH values. It will also be tried to improve the cyclic behavior of concrete by utilizing epoxy resin. In the following, a literature review in this field is discussed and the most important achievements of other studies are mentioned.

Golestaneh et al. studied the chemical resistance of PC samples in different solutions [9]. In this research, three different sizes of silica powder were used to make polymer concrete. Also, different concentrations of aggregate presence in PC, including 15, 30, and 60%, were considered. Moreover, acetic acid, citric acid, sodium hydroxide, hydrochloric acid, and sulfuric acid with different percentages were used for the corrosive solution. They reported that the compressive strength of the samples exposed to acetic acid decreased significantly. In addition, in relation to this environment, it has been observed that the weight of the sample decreases by 31%. But in general, polymer concrete has shown good resistance against other corrosive environments. Abdel-Fattah and El-Hawary investigated the flexural behavior of unreinforced and polymer reinforced concretes based on the four-point bending test [10]. They used three types of polymer resin, including epoxy from two different manufacturers and one type of polyester, with different percentages of polymer in the concrete mix, i.e., 9, 12, and 15%. The results showed that the flexural behavior and compressive strength of polymer concrete is much better than unreinforced concrete. In this regard, the main focus of most studies has been on investigating the behavior of unreinforced and reinforced concretes in the vicinity of fresh air. Of course, it is rare to find articles that investigate the effect of temperature changes [11,12,13,14] or moisture levels [15,16,17] on the mechanical properties of concrete. Even in terms of extracting fatigue properties of this vital material in construction industry (building construction, dam construction, etc.), there has been little favor, but the results obtained are valuable. In general, based on laboratory results published by world scientists, it can be stated that many factors have an effect on the fatigue behavior of concrete (ordinary or reinforced concretes), the most important of which are loading frequency, loading amplitude, rate of strain, specimen size, temperature, and environmental conditions [18]. For example, Woelfl et al. utilized a three-point bending fatigue test to study the influence of loading frequency on the fatigue properties of polymer concrete [19]. Chen et al. studied the impacts of loading frequency and stress amplitude on the low-cycle fatigue (LCF) characteristics of concrete in tension–tension mode [20]. They reported that fatigue life decreases with increasing stress amplitude under constant loading frequency mode. They also stated that the loading frequency has a significant effect on the fatigue properties of concrete. In this way, reducing the loading frequency leads to reducing the fatigue life of concrete under cyclic tension loading. Moreover, Medeiros et al. experimentally evaluated the fatigue properties of ordinary and reinforced concretes under compression cyclic loading and considering different loading frequencies [21]. Bedi et al. predicted the strength of glass-reinforced polymer (GRP) concrete and furthermore evaluated their fatigue behavior and derived the bending fatigue–life (S-N) diagrams [22]. In addition, he and his research team focused on the design of polymer concrete reinforced with polypropylene fibers from the viewpoint of the fatigue phenomenon [23]. Recently, in March 2023, Liu et al. published a comprehensive review article focusing on the material properties of ordinary concrete due to the destructive phenomena of fatigue and corrosion simultaneously [24]. In this article, they examined three scenarios, including experimental procedures to investigate the fatigue–corrosion behavior of concrete, the failure mechanism of conventional concrete under the simultaneous effects of corrosion and fatigue, and finally, fatigue damage models that are able to include corrosion effects in concrete durability evaluation. They reported that it is very difficult to perform fatigue and corrosion tests at the same time, and fatigue tests are usually performed after corrosion tests. In other words, the effects of corrosion can be observed in fatigue tests. This process is the most common method used by researchers, although it differs from reality. In addition, they stated that the effects of the corrosion phenomenon on the fatigue resistance of concrete are much greater than its effects on the static strength. Wang et al. investigated the behavior of concrete in the vicinity of hydrochloric acid mist as a corrosive industrial environment [25]. They discussed the corrosion mechanisms of concrete, and also presented the strength degradation law of concrete characterized by acidification depth and pH value. Shin and Yoo studied the effects of crack width caused by corrosive environment as well as the duration of exposure to the corrosive environment on the tensile properties of reactive powder concrete [26]. To conduct this study, they used a standard solution of 3.5% sodium chloride (NaCl). They reported that the sample with a pre-crack width of 0.02 mm and exposed to a corrosive environment for 20 weeks had little change in strength, but if the pre-crack width was 0.05 mm, the change in strength would be significant. Shao et al. predicted the service life of reinforced concrete piles under the influence of the corrosion–fatigue phenomenon with regard to marine environments [27].

Despite the value and reliability of the experimental results, the two parameters of cost and time consumption of the tests lead to a reduction in the scope of various parameters. This fact shows itself colorfully when dealing with a complex system, including a test specimen with complex geometry or a complex testing process. Here, the subject of fatigue–corrosion is one of the most complex phenomena, which is very difficult and, in some cases, impossible to test in accordance with reality. On the other hand, the development of science and simulation software has made it possible to study the behavior of complex systems with a little simplification and at a much cheaper cost than performing tests [28]. For example, in the field of investigating the behavior of concrete, Song et al. presented a new finite element (FE) model based on three-dimensional particles to estimate the lifetime of concrete under compression cyclic loading [29]. They validated the proposed model using the technique of comparing FE results with experimental results and claimed that the proposed model can well evaluate the behavior of the material under static, fatigue, and creep loads. De Maio et al. studied the damage influence on the modal properties of fiber reinforced polymer (FRP) concrete [30]. For this purpose, they presented a new numerical model based on quasi-static loading conditions. They used the pre-defined damage in the model and finally extracted the dynamic response in terms of natural frequencies and compared it with the experimental results.

As is clear from the articles mentioned above, valuable studies have been conducted in the field of simulating the behavior of reinforced concrete and practical results have been published, which are not within the scope of this article, and in this research, the investigation is performed only in laboratory conditions. Furthermore, from the literature review, it is clear that most of the laboratory evaluations were performed on different types of concrete in the vicinity of fresh air. This is despite the fact that the use of this material, i.e., concrete, is not only for exposure to fresh air, and sometimes they are placed in harsh working conditions and corrosive environments, so the estimation of their service life in such environments should be conducted correctly. Hence, the author tried to evaluate the fatigue properties of conventional and polymer concretes exposed to various corrosive environments with different pH values in vitro.

## 2. Materials and Specimens

### 2.1. Raw Materials

**Figure 1:**
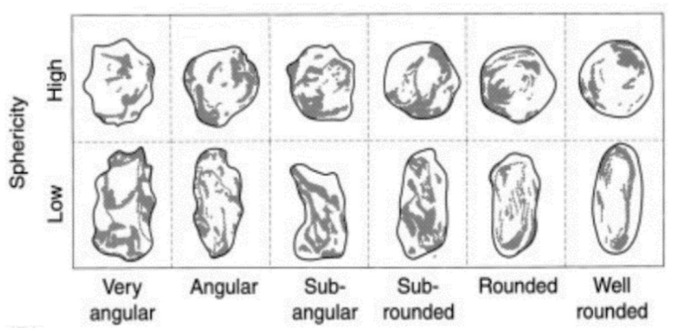
Aggregate classification in terms of appearance shape and geometry. Reprinted from ref. [1].

**Figure 2:**
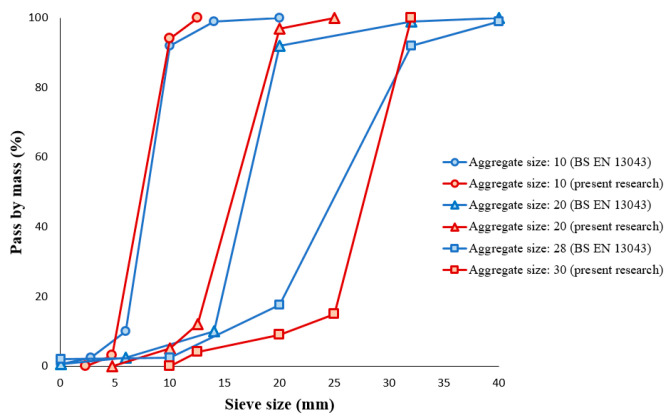
The sizing distribution of prepared aggregates compared to the standard. Reprinted from ref. [1].

To make concrete as a composite material, two different sizes of rounded aggregates, i.e., fine and coarse, were used in both CC and PC modes. In this regard, the classification of aggregates in terms of appearance and geometry is shown in Figure 1 [1]. The criterion considered for the fineness and coarseness of the aggregates is the average size of 4.75 mm. For this purpose, the BS EN 13043 standard was used to separate aggregates based on size, which must pass through sieves with different grades [31]. In this regard, the distribution of prepared aggregates compared to the standard is illustrated in Figure 2. In the following, aggregates with a size of 4.75 and smaller were called fine, aggregates with a size larger than 4.75 to 12.5 were called coarse, and aggregates with a size larger than 12.5 were removed from the test program in this research.

Another member of this composite material is adhesive, for which cement is used in conventional concrete. In the present research, Portland cement with specifications of Table 1 was used.

Epoxy resin was also used to make PC. In this way, EI-413 resin and HA-94 hardener were mixed with a ratio of 1:0.6. Finally, the technical specifications of the obtained epoxy resin are listed in Table 2.

### 2.2. Specimens

**Figure 3:**
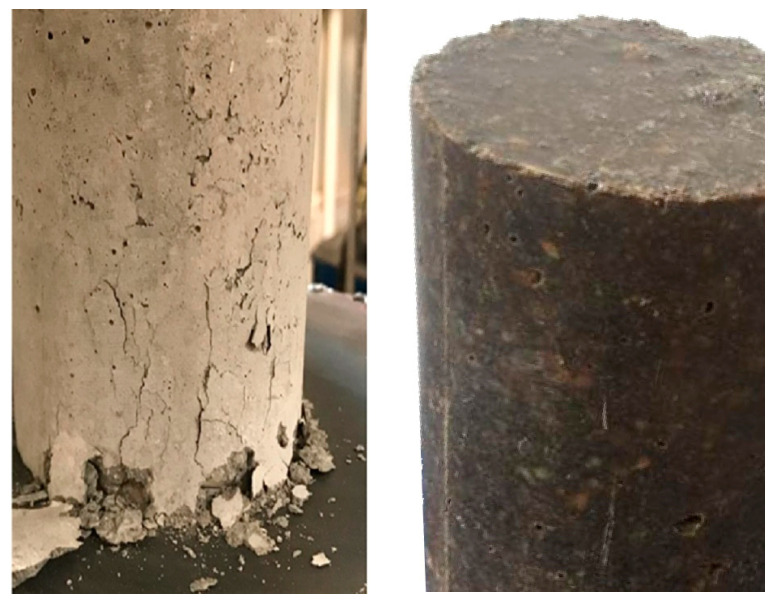
The provided laboratory samples, including conventional concrete with light color in the left side and polymer concrete with dark color in the right side.

In construction standards, two types of concrete samples are defined, namely cylinder and rectangular cube, whose laboratory results can be converted to each other with specific coefficients. In this research, laboratory samples were made in the form of cylinders with dimensions according to the ISO 1920-3 standard [32]. The diameter and height of the cylinders are 76 mm and 152 mm, respectively. In addition, in order to choose the proportions of concrete elements, the recommendations of the American Concrete Institute (ACI-211.1-91) was considered [33]. For CC, 30% fine aggregate, 46% coarse aggregate, and 17% cement were mixed with a water–cement ratio of 0.42. In the last step, curing was carried out at 20 degrees for 28 days. For PC, the proportion of coarse and fine aggregates was considered equal (44% for each) and 12% of epoxy resin was added to them. This means that PC samples do not contain cement. To this end, resin and polyamide as a hardener were mixed together for 5 min to obtain a homogeneous mixture. Then, the aggregates were added to the mixture through a mixer so that all the aggregates were covered by the epoxy resin and a homogenous reinforced concrete sample is created as much as possible. Finally, curing was performed at room temperature for 6 days. Due to the fact that this type of concrete, i.e., PC, does not contain cement and also water, it does not need a long time to dry and evaporate water. A total of 100 laboratory samples were made, half of which were CC, and the rest were PC. The proportion of their components is reported in Table 3. Figure 3 illustrates the provided samples. The light-colored samples are CC, and the PC is dark.

## 3. Experimental Work

As mentioned in the previous sections, the main objective of this article is to investigate the high-cycle fatigue behavior of conventional concrete and polymer concrete under the effect of corrosive environments. Therefore, before performing any mechanical tests, it is necessary to immerse the samples prepared in the previous step in different corrosive environments with different pH values. Thus, in the following, each of these subsections will be discussed separately.

### 3.1. Conditions of Corrosive Environments

To achieve the goals defined in this research, five different corrosive environments were considered. The first environment is the fresh air that most buildings are exposed to. Water was considered the second case. In this way, distilled water with pH of 7 was used to simulate rainy and foggy weather conditions. After that, sea water was considered. To simulate marine environments, making pH of 7.25 was attempted by adding different elements, such as calcium dichloride, magnesium dichloride, and sodium chloride, to distilled water. In addition, to simulate an acidic environment such as the behavior of acid rain, reducing the pH of distilled water from 7 to 2.5 was attempted by adding hydrochloric acid. And the last environment is dedicated to the alkaline environment. Normally, the pH of such environments is very high. Therefore, at first, increasing the pH value of distilled water was attempted by adding calcium hydroxide. As a result, the pH value became about 10. For further increase, sodium hydroxide was also added to the mixture to bring the pH value to 12.5. Despite the preparation of different environments, the pH values were checked weekly so that they did not lose their properties and remained within the defined range, otherwise the environment was changed. Ten samples of each type of concrete, i.e., CC and PC, were immersed in each of the environments for one month and then kept in the fresh air to dry. Now they are ready to perform various mechanical tests.

### 3.2. Mechanical Tests

#### 3.2.1. Compression Test

**Figure 4:**
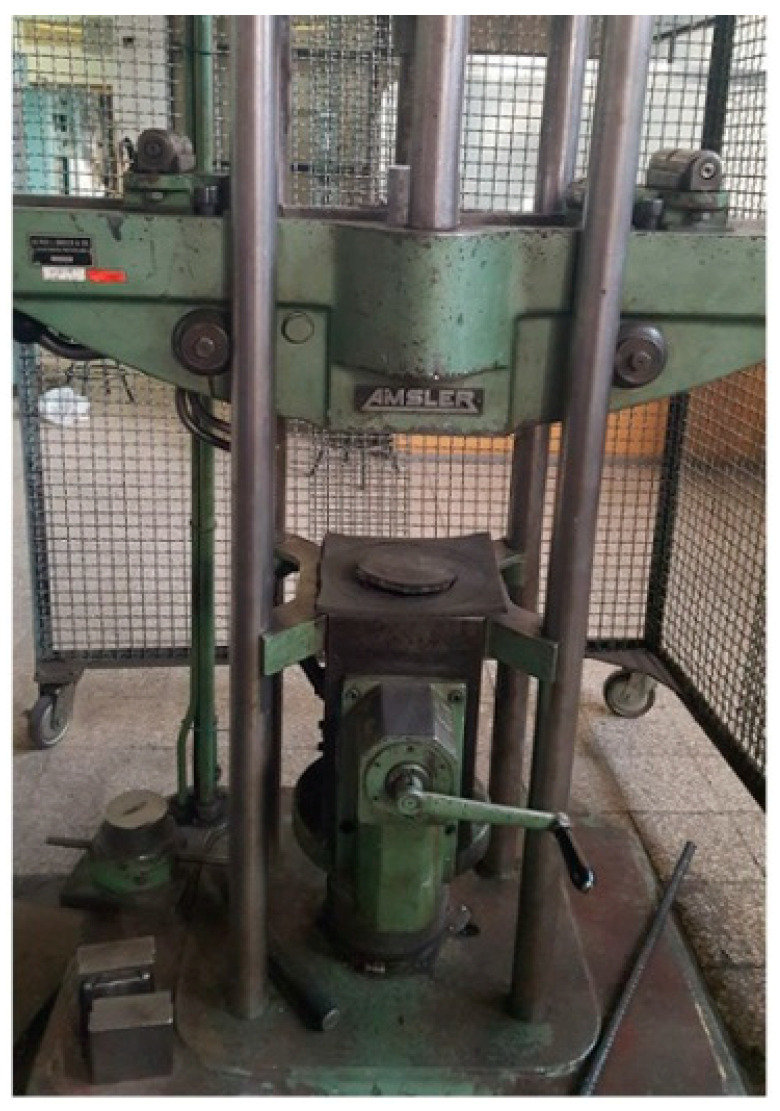
The employed static test facilities. Reprinted from ref. [2].

In order to determine the appropriate range of cyclic loading in the fatigue test, a compression test was performed on the samples. This test was performed according to the ISO-1920-4 standard [34] on both CC and PC samples considering the conditions stated in Section 3.1. For this purpose, as shown in Figure 4, hydraulic universal compression testing machine (Amsler brand, made in Acworth, GA, USA) with a capacity of 60 tons was employed. A constant loading rate of 1000 N/s was applied continuously without shock.

#### 3.2.2. Uniaxial Constant Amplitude Fatigue Test

Axial fatigue test was performed on 9 samples of each group of materials. All experiments were performed under constant amplitude and force-controlled conditions at room temperature. In this regard, the loading ratio, i.e., the ratio of minimum force to the maximum force, was considered equal to 0.1 and loaded with a frequency of 3 Hz. This means that the specimens are always under compressive load. Also, to apply force uniformly on the cross-sectional surface of the samples, 100 mm thick St52 plates were used at the beginning and end of the samples between the jaws of the testing machine. Moreover, to obtain the fatigue behavior in the high-cycle area and extract the coefficients of Basquin linear relationship, the tests were loaded at three different force levels, and the average number of cycles until the final failure of the sample and its collapse was reported as the number of fatigue cycles (each test was performed three times to check the repeatability of the response). During the tests, it was observed many times that a surface crack is initiated in the sample, and despite the long length of cracks even more than half the height of the sample, or the presence of deep cracks up to the depth of about 1/3 of the diameter of the sample, the sample can withstand cyclic load. Therefore, the test ends when the sample cannot withstand any force.

## 4. Results and Discussion

The static test results are reported in Table 4. It is clear that the static strength of polymer concrete has increased by about 12% compared to the strength of conventional concrete. Moreover, it can also be seen that the static strength reduction rate corresponding to different corrosive environments in polymer concrete is much lower than conventional concrete. This means that in all cases, PC is superior to CC.

**Figure 5:**
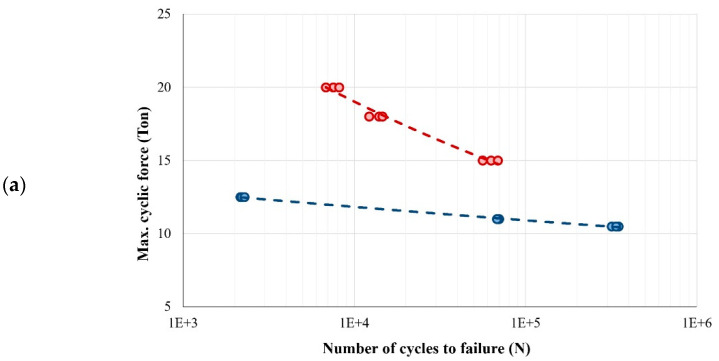

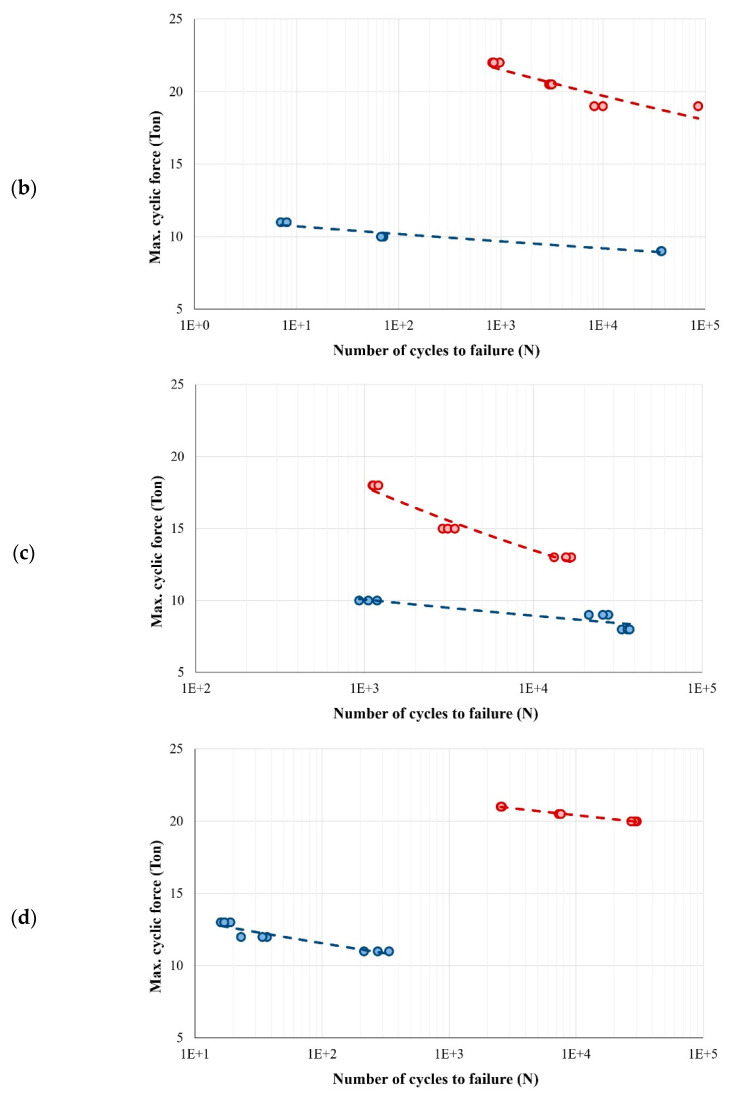

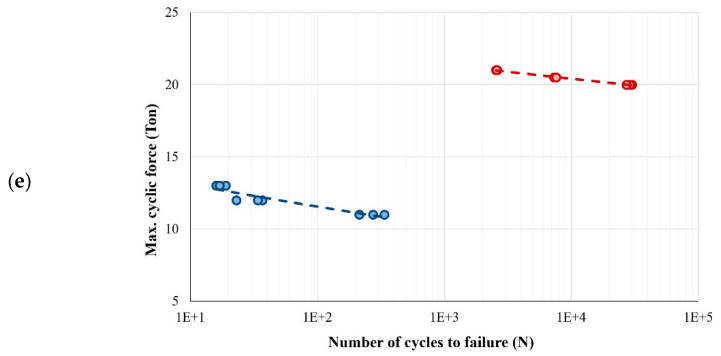
High-cycle region of S-N curve for CC and PC samples in different corrosive environments, including (**a**) exposed to fresh air, (**b**) exposed to W, (**c**) exposed to SW, (**d**) exposed to AC, and (**e**) exposed to AL.

Figure 5 shows the comparison of the fatigue behavior of CC and PC samples in different corrosive environments. The results are shown in two separate colors, blue and red, assigned to CC and PC samples, respectively.

**Figure 6:**
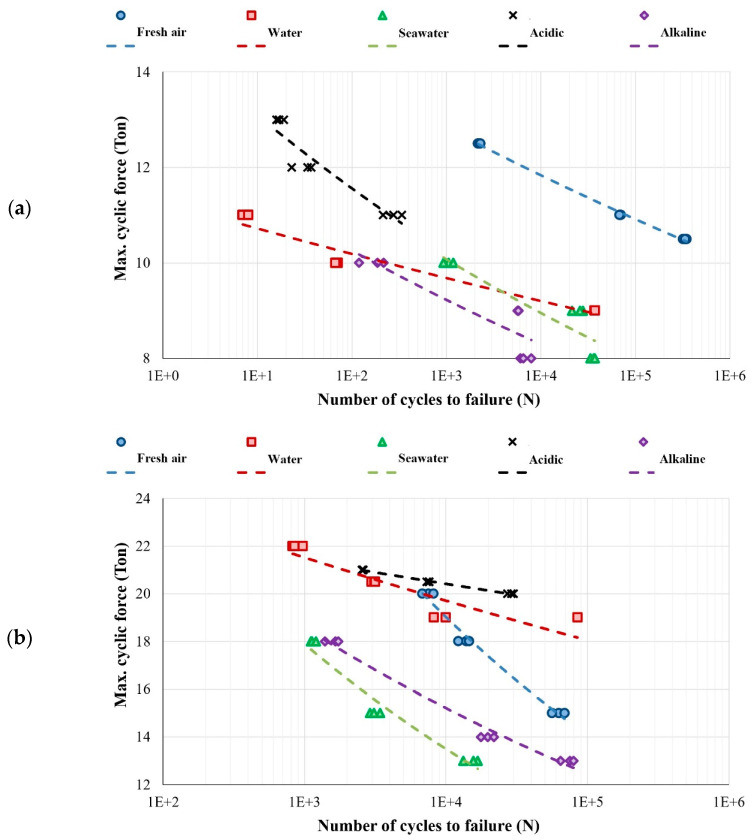
Comparison of S-N diagrams related to different environments with different pH values for (**a**) conventional concrete and (**b**) polymer concrete.

As is clear from the results presented in Figure 5, the fatigue properties of polymer concrete are much higher than the fatigue properties of conventional concrete. This result was observed in all corrosive environments and could be qualitatively predicted from the results of static tests. Furthermore, the results show that the change in the number of cycles to failure of CC samples is almost linear. Meanwhile, in polymer concrete, the changes in the number of cycles to failure are significant and linear with a steep slope. Also, the range of loads applied for the purpose of checking HCF is about 8 to 12 tons for conventional concrete, but it is about 18 to 22 tons for polymer concrete. In addition to the above-mentioned cases, it was observed in some results that with this loading range, the failure mode changed from the HCF region to the LCF region. In other words, the corrosive environment has weakened the concrete and is empty from the inside. For example, look at Figure 5d, which corresponds to the acidic environment (even the number of cycles to failure reaches a few fingers). Next, in order to compare the effect of each corrosive environment on the fatigue life of CC and PC samples, the S-N curves are shown in Figure 6a and Figure 6b, respectively. It can be seen in Figure 6a that the maximum fatigue life of conventional concrete is when it was in the vicinity of fresh air. Also, the lowest fatigue life of CC samples is when they are exposed in the AL environment. To better understand this matter, consider the changes in fatigue life as an example of a 10-ton load; also, all S-N curves are linear and the number of cycles to failure is in a logarithmic scale. In this case, the fatigue life of CC sample exposed to fresh air will be about one million cycles. It is about 200 cycles considering water environment. When it is in the sea water environment, it is about 1000 cycles. In the case of the AC environment, the number of cycles to failure is fewer than 1000. Finally, in the case of AL environment, the number of cycles to failure is about 100. However, this statistic is not accurate, and to obtain the exact number of cycles, the coefficients of Basquin equation should be extracted using the laboratory results and regression statistical data method. However, we will have a qualitative view of the matter. In addition, it can be seen that the highest negative slope is related to acidic, alkaline, and seawater corrosive environments, respectively. On the other hand, as the loading rate decreases and enters the very high-cycle fatigue (VHCF) state, the effects of these corrosive environments become more apparent. However, qualitatively, at a maximum cyclic force of 10 tons, it can be concluded that the fatigue life of CC sample in fresh air is approximately 10,000 times longer than when is in an alkaline environment. Also, its fatigue life is almost a thousand times when the CC sample is in an acidic environment or sea water. Finally, the lowest slope of fatigue life changes is related to CC samples in a fresh water environment.

From Figure 6b, it can be seen that the lowest fatigue life of polymer concrete sample is when it is in the vicinity of sea water and alkaline environment, respectively. But the noteworthy point here is that the PC samples in the vicinity of fresh air do not have the longest fatigue life, and it is likely that there were internal defects in the polymer concrete samples, because the longest fatigue life has been reported in acidic environment and then in the vicinity of fresh water. It can also be seen that the loading range is about 13 to 22 tons, and all the tests are related to the HCF area, i.e., more than 1000 cycles, which indicates the high strength of polymer concrete samples compared to conventional concrete samples. In addition, it was observed that the slope variations in polymer concrete samples in the vicinity of fresh air are much higher than in other corrosive environments. Now, for a qualitative comparison, consider the loading value of 18 tons; the fatigue life of the polymer concrete sample in the vicinity of fresh air, water, sea water, acidic, and alkaline environments is about 15,000, 100,000, 1000, 1,000,000, and 1500 cycles, respectively. In short, the author believes that more experiments are needed to make a general conclusion and to generalize it to other conditions, and due to the limitations of time, budget, and laboratory equipment (i.e., facilities), these results are good and usable for the development of preliminary mathematical models.

## 5. Conclusions

In this study, the author has tried to investigate the effect of various types of corrosive environments with different pH values on the fatigue properties, i.e., the life span under cyclic loading, of conventional and polymer concretes. In this regard, the research was completely experimental. To this end, different environments such as fresh air, water, sea water, acidic, and alkaline were used and the samples were evaluated by fatigue test after one month immersion in these environments. Finally, the S-N diagrams of different samples were compared with each other. Among the most important achievements of this research, the following can be mentioned:

1. The static test results showed that the compressive strength of polymer concrete is about 12% higher than the compressive strength of conventional concrete (both samples were in the vicinity of fresh air). Also, the reduction rate of the static strength of polymer concrete was less than conventional concrete in all the studied corrosive environments.
2. The fatigue resistance of polymer concrete is much higher than conventional concrete. This was proven in all corrosive environments. Also, in some cases, it has been shown that this increase in strength sometimes reaches 100%.
3. The fatigue test results on conventional concrete samples indicated that the longest fatigue life is related to the samples in the vicinity of fresh air. Also, the lowest fatigue life is related to samples immersed in an alkaline environment.
4. Fatigue test results on polymer concrete samples showed that the lowest fatigue life is related to the samples in the vicinity of water and alkaline environments.
5. Fatigue behavior of conventional and polymer concretes considering the conditions stated in this research was qualitatively and quantitatively discussed. In future study, the author plans to perform more experiments and analyses using statistical tools such as data mining techniques and machine learning tools. In addition, attempts are made to remove the current laboratory limitations, including equipment, so that the fatigue behavior can be investigated in two separate areas, i.e., low-cycle and high-cycle.

## Figures and Tables

**Table 1:** Specifications of Portland cement used in this research.

Blain	Setting Time Initial	Setting Time Final	Soundness	Autoclave Expansion	Compressive Strength
cm^2^/g	min	min	min	%	MPa
3250–3450	90–110	160–190	0.5–1.4	0.10–0.25	18–20

**Table 2:** The technical specifications of the obtained epoxy resin.

Compressive Strength	Corresponding Strain	Tensile Strength	Water Absorption	Glass Transition
MPa	%	MPa	%	℃
54.77	5.65	41.62	0.1	90

**Table 3:** The proportion of concrete components used in this research.

Concrete Type	Aggregate		Cement	Water/Cement Ratio	Epoxy Resin
	Fine (%)	Coarse (%)	%	%	%
Conventional	30	46	17	0.42	0
Polymer	44	44	0	0	12

**Table 4:** Compressive strength of conventional concrete and polymer concrete after one month immersion in different corrosive environments (all values are in MPa).

Corrosive Environment	Compressive Strength	
	CC	PC
Fresh air	44	49.6
Water	36.8	48
Sea water	34.6	45.9
Acidic	36.4	47.5
Alkaline	39.8	47

## Data Availability

The data that support the findings of this study are available from the corresponding author upon reasonable request.
